# Identification of potential drug-induced neuralgia signals through disproportionality analysis of the FAERS database

**DOI:** 10.3389/fphar.2025.1645114

**Published:** 2025-07-30

**Authors:** Yating An, Ying Zheng, Ziwei Jiang, Meng Meng, Jintuo Yin, Yahui An

**Affiliations:** ^1^ Department of Pharmacy, Beijing Health Vocational College, Beijing, China; ^2^ Department of Pharmacy, Fourth Hospital of Hebei Medical University, Shijiazhuang, Hebei, China; ^3^ Department of Pharmacy, Beijing Jishuitan Hospital, Capital Medical University, Beijing, China

**Keywords:** neuralgia, adverse drug event, FAERS database, neurotoxicity, signal detection

## Abstract

**Background:**

Drug-induced neuralgia is a common and significant adverse reaction. This study analyzed the United States food and drug administration adverse event reporting system (FAERS) database (2004–2024) to identify relevant drugs and potential mechanisms.

**Methods:**

We conducted an association analysis between drugs and neuralgia using the FAERS database. Disproportionality analysis methods, including the reporting odds ratio (ROR), proportional reporting ratio (PRR), Bayesian confidence propagation neural network (BCPNN), and empirical Bayesian geometric mean (EBGM), were applied. Data from 2004 to 2024 were analyzed to identify drugs potentially associated with neuralgia.

**Results:**

Among the 103,678 reports of neuralgia-related adverse events, 60.29% involved female patients, and 30.40% were aged between 41 and 64 years. The most common underlying medical conditions were plasma cell myeloma (14.28%) and multiple sclerosis (10.65%). The analysis revealed significant associations between neuralgia and several classes of drugs, including chemotherapeutic agents, certain antibiotics, and immunosuppressants, potentially attributable to neurotoxicity, immune-mediated mechanisms, or metabolic disruptions. Notably, lenalidomide exhibited the strongest association with neuralgia, followed by sodium citrate. These findings underscore the importance of early recognition, safer prescribing strategies, and further investigation to mitigate neurotoxic risks.

**Conclusion:**

This study identifies key drugs, including chemotherapeutics, antibiotics, and immunosuppressants, associated with drug-induced neuralgia through FAERS data analysis, highlighting the need for early detection, safer prescribing practices, and further research into mitigating neurotoxicity.

## 1 Introduction

Neuralgia is a type of pain resulting from damage or disease in the nervous system. The International Association for the Study of Pain defines neuropathic pain as pain caused by a lesion or dysfunction of the somatosensory system, which may involve the peripheral or central nervous system (International Association for the Study of Pain, 2025). Unlike nociceptive pain, which results from direct tissue injury, neuropathic pain originates from abnormal nerve signaling, often occurring in the absence of external stimuli (Bernetti et al., 2021). This type of pain can be persistent or episodic and may severely compromise the patient’s quality of life and psychological wellbeing.

Preventing neuralgia requires addressing underlying conditions, minimizing nerve injury, and ensuring rational drug use (Bannister et al., 2020). Although drug-induced neuralgia is relatively rare, it presents unique challenges owing to its often delayed onset and nonspecific symptoms (Yao et al., 2025; Merheb et al., 2022). Typically characterized by sharp, burning, or shooting pain, it may be misdiagnosed in its early stages, complicating timely intervention (Li and Liu, 2025). Among the various etiologies of neuropathic pain, chemotherapeutic agents are prominent contributors due to their neurotoxic effects (Etminan et al., 2014). Other recognized causes include certain antiviral agents and antibiotics (Han et al., 2023). Although treatments such as antidepressants, gabapentinoids, sodium channel blockers, TRPV1 agonists, and opioids are available, managing neuropathic pain remains challenging, due to interindividual variability in treatment response and an incomplete understanding of the underlying pathophysiological mechanisms (Baranidharan et al., 2023). Therefore, its pathophysiology underscores the critical need for further investigation (Jensen et al., 2011).

We conducted a pharmacovigilance study using data from the FAERS. FAERS serves as a vital resource in pharmacovigilance, facilitating the identification of potential drug-related adverse drug events (ADEs) through post-marketing surveillance. The database contains voluntarily submitted reports from healthcare professionals and consumers, offering valuable information on demographic characteristics, drug exposure, clinical outcomes, and more (Bousquet et al., 2005). Through systematic analysis of this database, we can identify disproportionality signals, which are statistical indicators suggesting a higher-than-expected frequency of a specific ADE associated with a particular drug (Fusaroli et al., 2024; Almenoff et al., 2007).

The objective of this study was to identify pharmacological agents potentially associated with drug-induced neuralgia by performing disproportionality analysis of the FAERS database. The findings aim to support clinicians in recognizing high-risk medications, inform future research directions, and ultimately enhance the management of neuralgia in patients receiving pharmacotherapy.

## 2 Materials and methods

### 2.1 Data source

This retrospective pharmacovigilance study was conducted using the FAERS database (https://fis.fda.gov/extensions/FPD-QDE-FAERS/FPD-QDE-FAERS.html). The FDA electronically processes adverse event reports and releases them quarterly in ASCII and XML formats, both of which are available for free download. We collected data from Q1 2004 to Q4 2024, as standardized FAERS quarterly datasets have been publicly available since 2004, and Q4 2024 represents the latest release at the time of our analysis. The extracted data covered seven components: DEMO (patient demographics and administrative information), DRUG (drug/biologic information), REAC (MedDRA terms for adverse events), OUTC (patient outcomes), RPSR (report sources), THER (drug therapy start/end dates), and INDI (MedDRA terms for diagnoses/indications).

### 2.2 Definition of ADEs and drugs

We included FAERS reports from Q1 2004 to Q4 2024 that involved human subjects, contained drug information in the DRUG file, adverse event terms in the REAC file, and basic demographic data in the DEMO file. To avoid redundancy, we applied the FDA-recommended deduplication strategy by retaining only the most recent version of each unique “CASEID” based on the “FDA_DT” field. We also excluded incomplete reports lacking drug identifiers, adverse event terms, or containing implausible dates (e.g., event onset before drug administration). We standardized all ADEs using preferred terms (PTs) from the Medical Dictionary for Regulatory Activities (MedDRA, version 26.1). To identify cases of neuralgia, we searched for the PT “neuralgia” (MedDRA code: 10029223) in the REAC table. Specific PTs are listed in Supplementary Table S1.

For drug identification, the generic name of each medication was used as a unique identifier. Since many reports included brand names, these were translated to generic names via the DrugBank database (https://go.drugbank.com/drugs). Reports containing drug names unavailable in DrugBank were deemed invalid and manually excluded from the analysis. Details of the data cleaning process are provided in Supplementary Figure S1.

Furthermore, we analyzed the time-to-onset (TTO) of adverse events for drugs with valid signals, defined as the interval between EVENT_DT and START_DT. Reports with date errors (e.g., EVENT_DT before START_DT), missing, or invalid values were excluded. TTO was assessed using medians, interquartile ranges, and the Weibull shape parameter.

### 2.3 Statistical analysis

Descriptive analyses were performed to summarize the clinical characteristics of patients with drug-induced neuralgia, including age, sex, indication, outcome, and reporting country. The top 30 drugs associated with neuralgia, based on report dates, are presented in Supplementary Table S2.

To explore potential associations between drugs and neuralgia, we conducted disproportionality analyses. This method compares the observed and expected frequencies of drug-AE pairs to detect significant imbalances. We applied four widely accepted algorithms: reporting odds ratio (ROR), proportional reporting ratio (PRR), Bayesian confidence propagation neural network (BCPNN), and empirical Bayesian geometric mean (EBGM) (van Puijenbroek et al., 2002; Huang et al., 2014). The equations and signal detection criteria for each method are detailed in Tables 1, 2.

**TABLE 1:** The 2 × 2 cross-table of ROR.

Counts of reports	Drug(s) of interest	All other drugs	Total
Adverse event(s) of interest	a	b	a + b
All other adverse events	c	d	c + d
Total	a + c	b + d	a + b + c + d

**TABLE 2:** Summary of algorithms used for signal detection.

Method	Formula	Threshold
ROR	$ROR=\frac{ad}{bc}$	a ≥ 3 ROR ≥ 3 95%Cl (lower limit) > 1
	$SE\left(lnROR\right)=\sqrt{\frac{1}{a}+\frac{1}{b}+\frac{1}{c}+\frac{1}{d}}$	
	$95\%CI=e^{\ln\left(ROR\right)\pm 1.96\cdot se}$	
PRR	$\text{PRR}=\frac{a/\left(a+b\right)}{c/\left(c+d\right)}$	a ≥ 3 PRR ≥ 2 95%Cl (lower limit) > 1
	$SE\left(lnPRR\right)=\sqrt{\frac{1}{a}-\frac{1}{a+b}+\frac{1}{c}-\frac{1}{c+d}}$	
	$95\%CI=e^{\ln\left(PRR\right)\pm 1.96\cdot se}$	
BCPNN	$IC=\log_{2}\frac{p\left(x,y\right)}{p\left(x\right)p\left(y\right)}=\log_{2}\frac{a\left(a+b+c+d\right)}{\left(a+b\right)\left(a+c\right)}$	IC025 > 0
	$E\left(IC\right)=\log_{2}\frac{\left(a+\gamma 11\right)\left(a+b+c+d+\alpha \right)\left(a+b+c+d+\beta \right)}{\left(a+b+c+d+\gamma \right)\left(a+b+\alpha 1\right)\left(a+c+\beta 1\right)}$	
	$V\left(IC\right)=\frac{1}{{\left(\ln2\right)}^{2}}\left[\frac{\left(a+b+c+d\right)-a+\gamma -\gamma 11}{\left(a+\gamma 11\right)\left(1+a+b+c+d+\gamma \right)}+\frac{\left(a+b+c+d\right)-\left(a+b\right)+a-\alpha 1}{\left(a+b+\alpha 1\right)\left(1+a+b+c+d+\alpha \right)}\gamma =\gamma 11\frac{\left(a+b+c+d+\alpha \right)\left(a+b+c+d+\beta \right)}{\left(a+b+\alpha 1\right)\left(a+c+\beta 1\right)}\right]$	
	$IC-2SD=E\left(IC\right)-2\sqrt{V\left(IC\right)}$	
EBGM	$\text{EBGM}=\frac{a\left(a+b+c+d\right)}{\left(a+b\right)\left(a+c\right)}$	EBGM05 > 2
	$SE\left(lnEBGM\right)=\sqrt{\frac{1}{a}+\frac{1}{b}+\frac{1}{c}+\frac{1}{d}}$	
	$95\%CI=e^{\ln\left(EBGM\right)\pm 1.96\cdot se}$	

To increase the specificity of signal detection, a signal was defined as positive only when the drug met the predefined threshold criteria across all four algorithms, indicating a robust association with neuralgia (Hosomi et al., 2018).

## 3 Results

### 3.1 Basic characteristics of adverse events related to neuralgia

**FIGURE 1:**
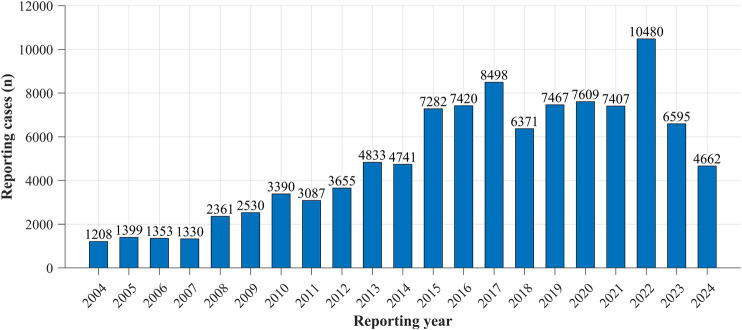
The number of reported cases of neuralgia from 2004 to 2024.

Reporting trends of drug-induced neuralgia cases from 2004 to 2024 were analyzed. As shown in Figure 1, the results revealed a general upward trajectory in the number of reports until approximately 2016, followed by fluctuating patterns in subsequent years. Notably, the number of reports peaked in 2022, representing the highest annual count observed during the entire study period.

The characteristics of 103,678 reported cases were summarized in Table 3, stratified by sex, age group, medical indication, clinical outcome, and reporting country. Among these cases, 60.29% were female, 31.42% were male, and 8.28% had unspecified sex information. Regarding age distribution, 36.55% of cases lacked age data. Among cases with available age information, individuals aged 41–64 years accounted for the largest proportion (30.40%), followed by those aged ≥65 years (17.18%), 19–40 years (14.04%), and <18 years (1.83%). Plasma cell myeloma was the most frequently reported indication, representing 14.28% (14,802 cases), followed by multiple sclerosis at 10.65% (11,044 cases). Other commonly reported indications included depression (3.25%), urinary tract infection (1.74%), and breast cancer (1.49%). Plasma cell myeloma and multiple sclerosis were the most prominent underlying conditions in the dataset. In terms of clinical outcomes, hospitalization was the most frequently reported severe event (12.81%), followed by disability (5.09%), death (2.15%), and life-threatening conditions (1.84%). Geographically, the majority of reports originated from the United States (73.92%), followed by the United Kingdom (4.81%), Canada (2.37%), France (2.04%), and Germany (1.97%).

**TABLE 3:** Clinical characteristics of reports with neuralgia.

Characteristics	Reports, n (%)
Sex	N = 103,678
Female	62,508 (60.29)
Male	32,592 (31.42)
Unknown	8,578 (8.28)
Age	
≤18	1,892 (1.83)
19–40	14,564 (14.04)
41–64	31,526 (30.40)
≥65	17,809 (17.18)
Unknown	37,886 (36.55)
Indications	
Plasma Cell Myeloma	14,802 (14.28)
Multiple Sclerosis	11,044 (10.65)
Depression	3,373 (3.25)
Urinary tract infection	1800 (1.74)
Breast cancer	1,544 (1.49)
Outcomes	
Death	2,229 (2.15)
Disability	5,282 (5.09)
Life-threatening	1,910 (1.84)
Hospitalization	13,276 (12.81)
Reported countries	
United States	76,613 (73.92)
United Kingdom	4,984 (4.81)
Canada	2,460 (2.37)
France	2,115 (2.04)
Germany	2,045 (1.97)

Despite substantial missing data, analysis of the FAERS database revealed three key trends. Females were predominantly represented in the demographic profile, the largest age subgroup to be 41–64 years and identified strong geographic clustering in the United States. Among the reported adverse outcomes, hospitalization and disability emerged as the most frequently documented events. Clinically, plasma cell myeloma and multiple sclerosis were the top-reported therapeutic indications in the dataset.

**FIGURE 2:**
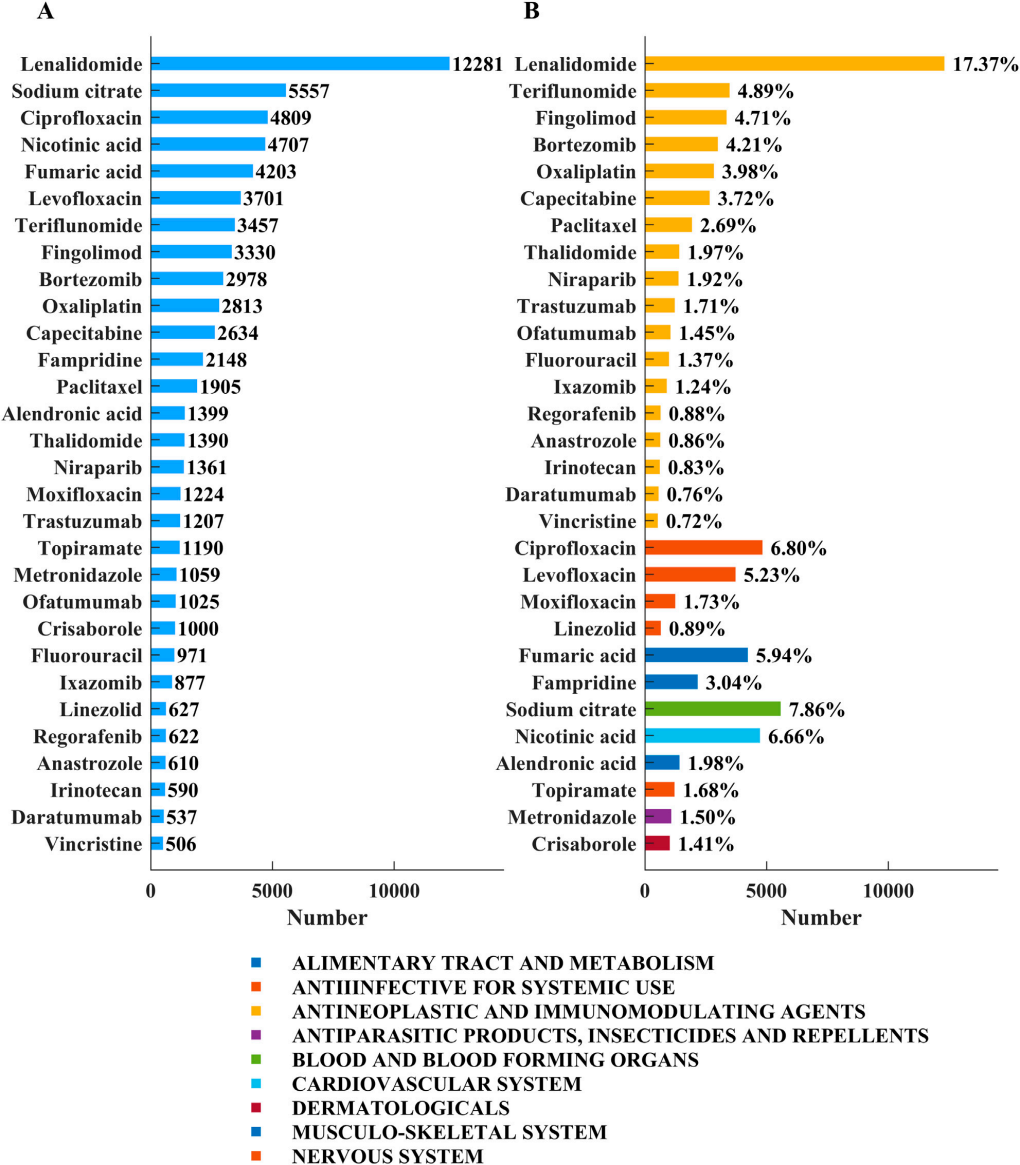
Top 30 medications with the most number of reported neuralgia cases. **(A)** The number of cases of the top 30 drugs. **(B)** The drug categories of the top 30 drugs and the percentage of total neuralgia events associated with each drug.

Drugs associated with the ADEs of neuralgia were ranked by the number of reported cases and are summarized in Figure 2. Figure 2A shows that lenalidomide was the most frequently reported agent, with 12,281 patients, followed by sodium citrate (5,557 patients), ciprofloxacin (4,809), nicotinic acid (4,707), levofloxacin (3,701), and teriflunomide (3,457). As shown in Figure 2B, antineoplastic and immunomodulatory agents accounted for the largest proportion of neurotoxicity‐related reports, with lenalidomide contributing 17.37%. Other frequently implicated drugs in this category included teriflunomide (4.89%), fingolimod (4.71%), and bortezomib (4.21%). Anti‐infectives such as ciprofloxacin (6.80%) and levofloxacin (5.23%) also exhibited high reporting rates. In addition, agents from other classes—including sodium citrate (7.86%), nicotinic acid (6.66%), and fumaric acid (5.94%)—were substantially represented. These findings indicate that neurotoxicity signals are particularly concentrated in antineoplastic agents, although a wide range of drug classes are involved. An analysis of Preferred Terms (PTs) related to specific types of neuralgia and the corresponding number of drug-related cases is provided in the Supplementary Figure S2.

### 3.2 Signal detection of adverse events related to neuralgia

The top 30 drugs associated with drug-induced neuralgia, listed in Table 4, were identified through disproportionality analysis using four signal detection algorithms: ROR, PRR, EBGM05, and IC025. In addition, time-to-onset analysis was conducted to evaluate the temporal pattern of neuralgia occurrence following drug exposure.

Sodium citrate (Rank #1) exhibited very high disproportionality values (ROR 300.86) with a short median TTO of 2 days, indicating rapid onset of neuralgia. Drugs with few reports, such as clobetasone butyrate (ROR 23.33, n = 4) and gadofosveset (ROR 18.23, n = 10), showed prolonged TTOs, suggesting delayed effects that warrant further investigation. Retapamulin (ROR 16.05, n = 143) had a median TTO of 2 days. More commonly reported drugs like nicotinic acid and ciprofloxacin demonstrated moderate disproportionality and relatively short TTOs of 9 and 4 days, respectively, indicating consistent and timely reporting.

**TABLE 4:** Top 30 drugs for signal strength of drug-induced neuralgia.

Ranking	Medication	n	ROR (95%Cl)	PRR (χ^2^)	EBGM05	IC 025	Package insert suggests risk for hypoglycemia	TTO (days)
1	sodium citrate	5,557	300.86	50.44	46.93	5.58	N	2 (1–4.75)
2	clobetasone butyrate	4	23.33	16.95	6.42	2.59	N	1708.5 (397–3,020)
3	gadofosveset	10	18.23	14.13	7.80	2.85	N	-
4	retapamulin	143	16.05	12.80	10.96	3.41	N	2 (1–16.5)
5	pyridoxine	19	14.03	11.50	7.56	2.82	Y	-
6	diclofenamide	284	13.49	11.14	10.00	3.29	N	13 (3–58.75)
7	lazertinib	4	12.28	10.32	4.19	1.95	Y	4.5 (2–22.75)
8	sertaconazole	5	11.22	9.57	4.30	1.97	N	-
9	azelaic acid	164	10.37	8.95	7.79	2.92	Y	5 (1.75–12.0)
10	nelarabine	80	10.37	8.96	7.34	2.82	Y	32 (10–59.75)
11	futibatinib	13	9.98	8.67	5.30	2.29	N	45.5 (14.0–56.75)
12	nicotinic acid	4,707	9.69	8.46	8.14	3.02	Y	9 (2–55)
13	crisaborole	1,000	9.10	8.01	7.55	2.90	N	4 (1–17.5)
14	ciprofloxacin	4,809	8.73	7.73	7.44	2.89	Y	4 (2–15)
15	tinidazole	8	8.48	7.53	4.05	1.89	Y	4 (1–11.5)
16	olsalazine	4	8.33	7.42	3.09	1.51	Y	-
17	amifampridine	210	8.18	7.29	6.46	2.66	Y	67 (4.25–395)
18	pyrithione	8	7.41	6.68	3.61	1.72	N	-
19	gadoversetamide	40	7.03	6.38	4.85	2.20	Y	57 (17.75–126.75)
20	enfortumab vedotin	305	7.02	6.38	5.77	2.50	Y	14 (7.5–53.5)
21	capreomycin	4	6.67	6.08	2.56	1.24	N	-
22	levofloxacin	3,701	6.62	6.05	5.82	2.53	Y	4 (2–12)
23	vinblastine	20	6.55	5.99	4.07	1.92	Y	-
24	repotrectinib	6	6.36	5.84	2.88	1.39	Y	-
25	monomethyl fumarate	73	6.16	5.67	4.63	2.15	N	70.5 (1–651.25)
26	bortezomib	2,978	6.14	5.65	5.43	2.43	Y	47.5 (14–98.75)
27	cerivastatin	3	6.03	5.56	2.06	0.95	Y	-
28	hexaminolevulinate	4	5.98	5.52	2.33	1.10	N	-
29	oxaliplatin	2,813	5.90	5.45	5.24	2.38	Y	42 (14–105)
30	teriflunomide	3,457	5.75	5.32	5.12	2.35	Y	119 (24.75–390.25)

Oncology agents, including nelarabine, bortezomib, and oxaliplatin, showed significant signals with median TTOs ranging from approximately one to one and a half months, underscoring the need for careful monitoring. Drugs with strong signals but limited cases, such as lazertinib and sertaconazole, exhibited short TTOs, highlighting the necessity for further validation.

Collectively, our analysis revealed a spectrum of disproportionality signals, ranging from extreme values observed with sodium citrate to potential early warning signs associated with less frequently reported agents such as clobetasone butyrate and gadofosveset. Notably, oncology and anti-infective drugs emerged as key therapeutic categories requiring continued pharmacovigilance. Drugs exhibiting strong signals but limited case counts should be prioritized for further investigation to substantiate.

## 4 Discussion

Neuropathic pain arises from various causes, including nerve trauma, compression, vascular and neurological diseases, infections, metabolic disorders, medications, and hereditary conditions (Jones et al., 2020). It is a complex condition resulting from nervous system damage or dysfunction and is commonly associated with diabetes mellitus, infections, and nerve trauma (Besi et al., 2015). However, certain medications can also induce neuropathic pain through mechanisms such as direct neurotoxicity, mitochondrial dysfunction, immune-mediated responses, and metabolic imbalances that impair nerve function (Zhuo et al., 2011). The severity and persistence of symptoms depend on multiple factors, including drug type, dosage, treatment duration, and patient susceptibility (Guo et al., 2024). Notably, a study in Taiwan identified renal disease as a predisposing factor for ethambutol-induced optic neuropathy, underscoring the role of underlying conditions in drug-induced neurotoxicity (Chen et al., 2012). Given its significant clinical impact, drug-induced neuropathic pain necessitates early recognition and intervention to minimize patient burden. A thorough understanding of the mechanisms and risk factors associated with neurotoxic medications enables clinicians to optimize treatment strategies while mitigating adverse effects. Future research should prioritize the development of neuroprotective approaches to prevent or reduce the risk of drug-induced neuropathy.

The results of this study indicate a continuous annual increase in adverse event reports related to drug-induced neuralgia, peaking in 2022. This trend may be attributed to multiple factors, including the growing use of medications, heightened public awareness of adverse reactions, regulatory and policy changes, advancements in medical technology, and improved data sharing and transparency (Baah, 2020). These factors collectively contribute to the rising number of reported cases. Furthermore, the findings suggest that the incidence of drug-induced neuropathic pain is significantly higher in females than in males. Although clinical observations do not indicate substantial sex-based differences in responses to analgesic medications, research on sex differences in neuropathic pain remains limited (Salis et al., 2024). Notably, variations in neuropathic pain intensity have been documented in diabetic patients, with females generally reporting higher pain sensitivity and intensity than males (Abraham et al., 2018). Moreover, sex-related differences may influence responses to certain analgesics, often necessitating dose adjustments or alternative pain management strategies (Marcianò et al., 2024). Hormonal fluctuations in females may further affect pain perception and treatment efficacy, highlighting the need for more targeted research in this area.

Lenalidomide, used to treat multiple myeloma and lymphoma, can cause peripheral neuropathy, though less frequently than thalidomide. Symptoms include numbness, tingling, and pain in the extremities (Cao et al., 2022). Its neurotoxicity may stem from mitochondrial dysfunction, oxidative stress, or immune-related mechanisms (Deng et al., 2021; Stino et al., 2024). Regular monitoring is essential, and dose adjustments or supportive treatments like vitamin B supplementation may help manage symptoms (Dalla Torre et al., 2016). Early detection and intervention can improve patient outcomes. Sodium citrate serves as an anticoagulant, preservative, and pH regulator, with widespread applications in both the food industry and medical field (Shvetsova et al., 2024). Studies have demonstrated that in sustained low-efficiency dialysis, sodium citrate provides effective regional anticoagulation without affecting systemic coagulation function, offering a viable alternative for clinicians (Zhang et al., 2013). Additionally, research indicates that using a 4% sodium citrate solution as a locking agent for central venous catheters in ICU patients is safer than heparin, as it reduces the risk of bleeding and catheter occlusion without increasing the incidence of hypocalcemia or infections (Deng et al., 2023). Furthermore, sodium citrate has been shown to inhibit cancer cell metabolism, suppressing proliferation and enhancing chemotherapy efficacy (Icard et al., 2024; Yin et al., 2025). It also improves immune cell function by increasing glucose availability, thereby promoting an antitumor immune response, suggesting its potential role as an adjuvant in immunotherapy (Wu et al., 2024).

Drug-induced neuralgia, a type of neuropathic pain resulting from medication-related nerve damage, is a critical concern in clinical practice (Si et al., 2018). Among the most commonly implicated drug classes are chemotherapeutic agents, antibiotics, and immunosuppressants, each of which causes nerve injury through distinct biological mechanisms (van Velzen et al., 2020). Understanding these mechanisms is essential for developing effective preventive and therapeutic strategies. Chemotherapeutic agents, particularly platinum-based drugs such as cisplatin and oxaliplatin, taxanes such as paclitaxel and docetaxel (Tian et al., 2025), and vinca alkaloids such as vincristine, are known to cause peripheral neuropathy (Ye and Abdi, 2025). These drugs induce nerve damage through mitochondrial dysfunction, oxidative stress, and microtubule disruption, leading to impaired neuronal function and persistent pain (Sisignano et al., 2014). Similarly, certain antibiotics, including aminoglycosides such as gentamicin and amikacin, polymyxins, and fluoroquinolones such as ciprofloxacin, have been associated with neurotoxicity (Kuehn, 2013). These antibiotics can disrupt neuronal membranes, interfere with ion channel function, and hinder neuronal repair, with the severity of neuralgia often correlating with the dosage and duration of treatment (Rezaei et al., 2018). Furthermore, immunosuppressants, particularly biologic agents targeting tumor necrosis factor-alpha, may contribute to neuropathic pain by altering immune responses and triggering neuroinflammation. Prolonged use can further impair nerve repair mechanisms and exacerbate neural damage, increasing the risk of persistent pain (Lallana and Fadul, 2011).

Overall, drug-induced neuralgia is a multifactorial condition driven by neurotoxic, immune-mediated, and metabolic disturbances. Recognizing the mechanisms by which different drug classes contribute to neuropathic pain is essential for minimizing risks, guiding clinical decisions, and improving patient outcomes.

The FAERS database plays a crucial role in drug safety research but has notable limitations. It relies on voluntary reporting, leading to reporting bias and underreporting of certain adverse events. The lack of exposure data prevents accurate risk estimation, and data quality issues, including missing or inconsistent information, further complicate analysis. FAERS cannot establish causal relationships due to potential confounding factors, and media influence may amplify reporting. Duplicate reports, delayed data updates, and limitations in adverse event classification also affect reliability. Additionally, most data come from the U.S. and other high-income countries, reflecting differences in healthcare systems and reporting habits. This may limit how well our findings apply to low- and middle-income countries and may not reflect the global burden of drug-induced neuralgia. We recommend future pharmacovigilance efforts include more diverse international data to improve fairness and accuracy.

## 5 Conclusion

This study analyzed the FAERS database to identify drugs associated with neuralgia. Chemotherapy drugs, certain antibiotics, and immunosuppressants were major triggers, likely causing nerve damage through neurotoxicity, immune responses, or metabolic disturbances. The findings indicate a higher prevalence in women. In terms of indications, multiple myeloma and multiple sclerosis were the most frequently reported. The study highlights the importance of early monitoring and rational drug use. These insights provide guidance for future research and clinical practice.

## Data Availability

The original contributions presented in the study are included in the article/Supplementary Material, further inquiries can be directed to the corresponding author.
