# Supplementary material for: Identification of potential drug-induced neuralgia signals through disproportionality analysis of the FAERS database
Source: Front Pharmacol. 2025 Jul 30;16:1645114. doi: 10.3389/fphar.2025.1645114 (PMC12343520; doi:10.3389/fphar.2025.1645114)
Supplement: Supplementary file 1 [file DataSheet1.pdf]

Supplementary Table 1. Neuropathic Pain-Related Preferred Terms (PTs) Associated with the Included Drugs

Neuralgia	Trigeminal neuralgia	Postherpetic neuralgia
Intercostal neuralgia	Occipital neuralgia	Phantom limb syndrome
Burning sensation	Sensory neuropathy hereditary	Allodynia
Dysaesthesia	Diabetic neuropathy	Neuropathy peripheral
Hyperalgesia	Glossopharyngeal neuralgia	Radicular pain
Paresthesia	Hereditary motor and sensory neuropathy	

Supplementary Table 2. Top 30 Drugs Associated with ADEs of Neuralgia

Lenalidomide	Sodium Citrate	Ciprofloxacin	Nicotinic Acid	Fumaric Acid
Levofloxacin	Teriflunomide	Fingolimod	Bortezomib	Oxaliplatin
Capecitabine	Fampridine	Paclitaxel	Alendronic Acid	Thalidomide
Niraparib	Moxifloxacin	Trastuzumab	Topiramate	Metronidazole
Ofatumumab	Crisaborole	Fluorouracil	Ixazomib	Linezolid
Regorafenib	Anastrozole	Irinotecan	Daratumumab	Vincristine

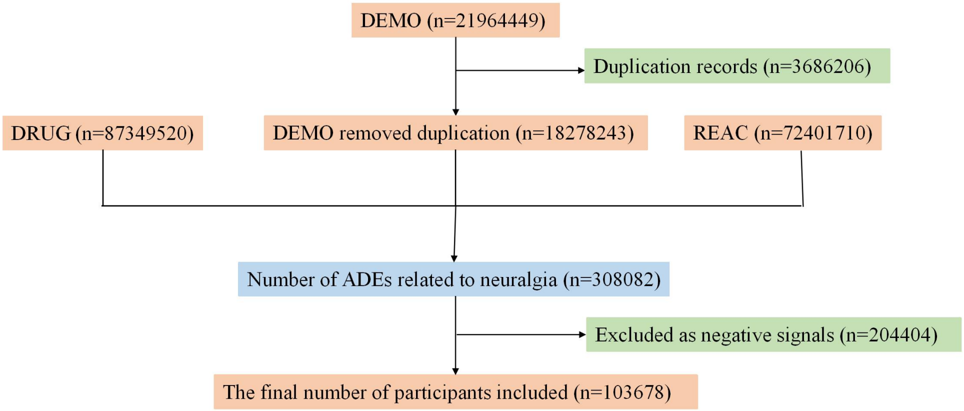

Supplementary Figure 1. Data Cleaning Flowchart for Drug-Induced Neuralgia

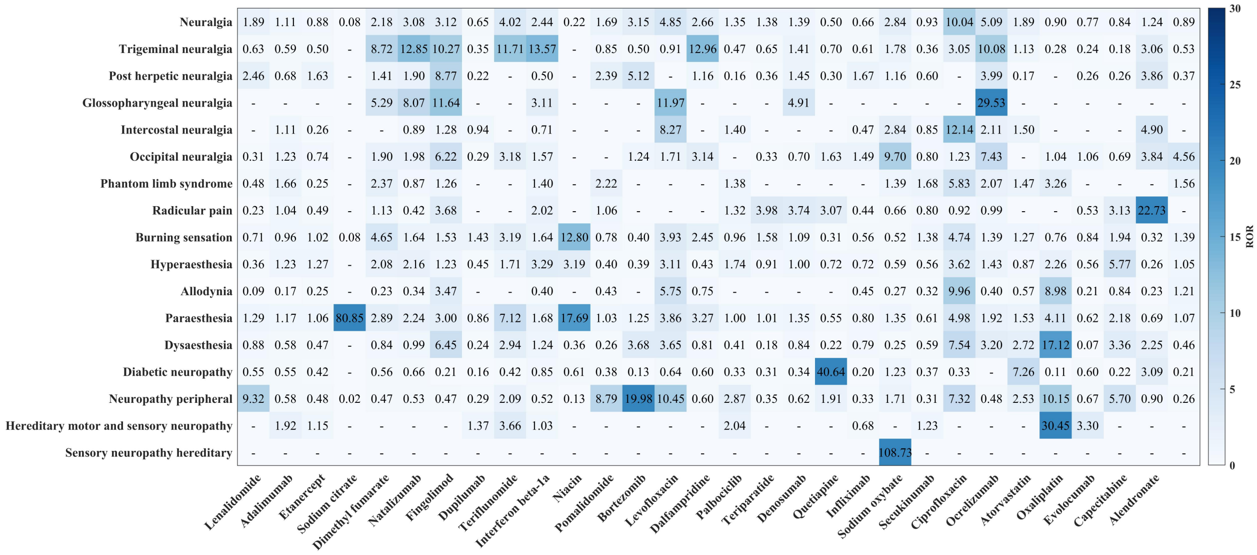

Supplementary Figure 2. Heat map of neuropathic pain-related Preferred Terms (PTs) and corresponding drug-associated adverse event case counts.
